# Association between autophagy and acute pancreatitis

**DOI:** 10.3389/fgene.2023.998035

**Published:** 2023-01-30

**Authors:** Tao Zhang, Yu Gan, Shuai Zhu

**Affiliations:** ^1^ Department of Pancreatic Surgery, Xiangya Hospital, Central South University, Changsha, China; ^2^ Department of General Surgery, Xiangya Hospital, Central South University, Changsha, China; ^3^ National Clinical Research Center for Geriatric Disorders, Xiangya Hospital, Changsha, China; ^4^ Department of Urology, Xiangya Hospital, Central South University, Changsha, China

**Keywords:** acute pancreatitis, autophagy, N6-methyladenosine, mechanism, advance

## Abstract

Autophagy pathway involves maintaining intracellular homeostasis by regulating the degradation of cytoplasmic components. Disfunction of autophagic process has been confirmed to be critical mechanism in many diseases, including cancer, inflammation, infection, degeneration and metabolic disorders. Recent studies have shown that autophagy is one of the early events in acute pancreatitis. Impaired autophagy promotes the abnormal activation of zymogen granules and results in apoptosis and necrosis of exocrine pancreas. Furthermore, multiple signal paths involve progression of acute pancreatitis by regulating autophagy pathway. This article provides a comprehensive review of the recent advances in epigenetic regulation of autophagy and the role of autophagy in acute pancreatitis.

## 1 Introduction

Acute pancreatitis (AP) is one of the most common gastrointestinal emergency events with varying clinical courses, ranging from self-limiting disorder to severe disease (Boxhoorn et al., 2020). Standard management of AP has been updated considerably in the past 10 years, the comprehensive and tailored treatments of multidisciplinary teams reduced both morbidity and mortality (Mederos et al., 2021). However, this unpredictable and potentially lethal disease remains a huge challenge for gastroenterologists owing to its complex and unclear pathogenesis.

To date, significant progress has been made in exploration of the pathophysiological mechanisms of AP. Acinar cell toxins and intraductal events can both trigger a series of intracellular responses including pathological calcium signaling, mitochondrial dysfunction, premature trypsinogen activation, endoplasmic reticulum stress, impaired unfolded protein response (Lee and Papachristou, 2019). However, deep insight and better understanding of molecular mechanism of acute pancreatitis are far away from well-illustrated.

Autophagy is a highly conserved decomposition process through which cytoplasmic materials such as damaged organelles and unwanted macromolecular substances can be degraded in the lysosomes, and the degradation products are recycled to maintain cellular homeostasis (Mizushima and Levine, 2020). The degradation phenomenon of intracellular components was firstly described by several scientists in 1950s and 1960s (Clark, 1957; Ashford and Porter, 1962; De Duve, 1963). In 1963, Christian de Duve named the degradation process as autophagy officially in the CIBI Foundation Symposium on Lysosomes (De Duve, 1963). Since then, numerous studies reported that the molecular pathway of autophagy has a universal and vital function in a wide range of human diseases, including cancer, inflammation, infection, neurodegeneration and metabolic disorders.

In recent years, accumulative researches have uncovered the relevance between AP and autophagy. The feature of autophagy in both experimental and human pancreatitis is the accumulation of vacuoles accompanied by increased LC3-II, p62 and decreased LAMP-2 (Helin et al., 1980; Koike et al., 1982; Mareninova et al., 2015). Studies proved that the vacuoles are mainly autophagosomes and autolysosomes, which are larger than that in basal and starvation-induced autophagy (Mareninova et al., 2009). Autophagy blockade through disruption of genes encoding ATG5, ATG7, LAMP-2 or IKK α stimulates activation in acinar cells of the proinflammatory transcription factors, such as NF-κB and STAT3, resulting in upregulation of cytokines and chemokines and inflammatory cell infiltration in the pancreas (Yang et al., 2016; Habtezion et al., 2019). Actually, abundant basal and starvation-induced autophagy has been confirmed to exist in mouse exocrine pancreas and be far more variable than in other organs (Mizushima et al., 2004). Basal autophagy maintains pancreatic acinar cell homeostasis and protein synthesis and prevents ER stress (Antonucci et al., 2015). Perspective from physiological function, pancreatic acinar cells secrete ample digestive enzymes and zymogens. Furthermore, In AP rodent models, lysosomal markers accumulate in the ZG-enriched subcellular fraction, which indicates that autophagy may play a role in regulating the fate of zymogen granules (Mareninova et al., 2009). In this review, we describe recent progress in the role and regulation of autophagy in AP. Additionally, we discuss the potential applications of autophagy signaling molecules in AP.

## 2 Process and regulation of autophagy pathway

Autophagic flux, the entire process of autophagy, mainly includes the origination of autophagosomes, the formation of autolysosomes and degradation of materials (Gukovskaya et al., 2017). In the process of autophagy, membrane dynamics is the core link initiated by autophagy-related genes (ATG). The hallmark of autophagy biogenesis is the formation of the double-membrane vesicular autophagosome. About 30 ATG proteins have been found involving the process of autophagosome biogenesis. There are six functional group of protein complex in mammal (Nakatogawa, 2020): (Boxhoorn et al., 2020) ULK complex; (Mederos et al., 2021); Atg9/ATG9-containing vesicles; (Lee and Papachristou, 2019); PI3K complex I; (Mizushima and Levine, 2020); ATG2–WIPI complex; (Clark, 1957); ATG16L1 complex; (De Duve, 1963); Atg8-family protein lipidation system (Mizushima and Levine, 2020). Subsequently, the membrane of autophagosome precursor will expand and form a closed-loop encapsulating the cytoplasm component. The lysosome then fuses with the outer membrane of autophagosomes and release many hydrolases to degrade the inner autophagosomal membrane and encysted materials (Levine and Kroemer, 2019).

In many diseases, autophagosome could be successfully generated through typical autophagy pathway, but the fusion of autophagosomes with lysosomes and degradation of substrate is impaired. This process is termed as incomplete autophagy flux (Zhang et al., 2022).

**FIGURE 1:**
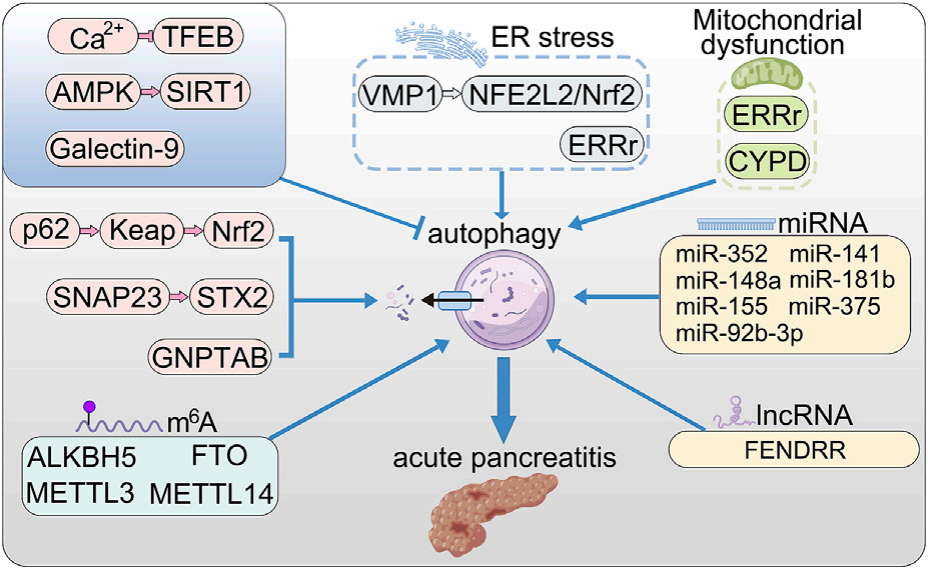
The roles of autophagy in AP. VMP1 promotes autophagy *via* NFE2L2/Nrf2 pathway; ERRγ promotes autophagy by reducing mitochondrial dysfunction and ER stress; Nrf2 promotes excessive autophagy through the p62–Keap1–Nrf2 signaling pathway; SNAP23 and STX2 promote autophagy by triggering SNARE complex; store-operated Ca^2+^ entry (SOCE) triggers calcium overload and activated TFEB *via* calcineurin activation, thereby enhanced autophagy; activation of AMPK relieved accumulation of autophagy by up-regulating SIRT1; Galectin-9 binds to Asn^175^ of Lamp2 and poly-LacNAc moieties to maintain lysosome function; long non-coding RNA FENDRR positively regulates autophagy through epigenetic suppression of ATG7 by binding PRC2; CypD maintains mitochondrial membrane and positively regulate lysosomal function and autophagy; microRNAs (miRNA) promote the initial stages of autophagy.

The nuclear regulatory network of autophagy is extremely complex (Figure 1). Recent studies have indicated that transcriptional control of autophagy plays a vital role in autophagy flux. Transcription factors, including FOXO family, E2F family, p53 and Ume6 complex, regulate the expression of ATG in different stages (Füllgrabe et al., 2014). It is currently recognized that TFEB and ZKSCAN3 are major antagonistic factors during autophagy. The nucleocytoplasmic translocation of TFEB significantly affects the biogenesis and function of lysosomes positively regulating autophagy, as well as upregulating autophagy genes including LC3 and SQSTM1 (Yan, 2022). Contrary to TFEB, several studies identified that ZKSCAN3 is the major transcriptional repressor of autophagy by targeting biogenesis and fusion of autophagosome and lysosome in cultured cells (Chauhan et al., 2013; Barthez et al., 2020; Pan and Valapala, 2021). However, *in vivo* mouse model, ZKSCAN3 did not serve anticipated effects on autophagy (Pan et al., 2017). One possible reason for this difference is that ZKSCAN3 may regulate autophagy by multiple mechanisms in different types of models or exist various regulatory pathways between normal tissue and tumor cells.

N6-methyladenosine (m6a) is associated with growth, occurrence and progression of disease and drug resistance of cancer cells. A study found that METTL3-mediated m6a methylation inhibited autophagy *via* decreasing stability of ATG5 mRNA to sustain porcine blastocyst development (Cao et al., 2021). In addition, scholars have provided evidence on the negative association between METTL14-mediated decreased autophagy and testosterone synthesis in Leydig cells, which indicated that m6a modification-mediated autophagy involved in body growth and development (Chen et al., 2021).

The effects of m6a modification on different diseases may be various by acting on diverse targets. METTL3 attenuates ATG7 mRNA stability in a YTHDF2-dependent manner and thus inhibits autophagy in osteoarthritis mouse models (Chen et al., 2022). Furthermore, the expression level of lysosomal protein Rubicon can be elevated by METTL3-mediated m6a modification, which inhibits the fusion of autophagosome and lysosome and then promotes the development of non-alcoholic fatty liver disease in mice (Peng et al., 2022). Similarly, in myocardial ischemia/reperfusion mouse model, METTL3 motivates RNA-binding protein HNRNPD to combine with TFEB pre-mRNA and subsequently restrains the expression of TFEB, while ALKBH5 plays an opposite role (Song et al., 2019). METTL14 promotes the translation of DNA damage-binding protein two and suppresses ultraviolet B radiation-induced skin tumorigenesis (Yang et al., 2021). METTL14 aggravates podocyte injury and glomerulopathy progression through m6a-dependent downregulating of Sirt1 (Lu et al., 2021). Knockdown of WTAP increased stability of LKB1 mRNA to decrease phosphorylation of AMPK, thereby promoting autophagy in hepatocellular carcinoma (HCC) (Li et al., 2021).

Furthermore, study has proved that FTO directly targets ATG5 and ATG7 mRNA in a YTHDF2-dependent way and positively regulate autophagy and adipogenesis (Wang et al., 2020a). Interaction has been confirmed between FTO and autophagy. In arsenic-associated human skin lesions, arsenic-mediated autophagy inhibition increased the stability of FTO proteins, and then accumulated FTO further inhibited autophagy through downregulating ATG5 and ATG7, increasing phosphorylation of AMPK, and decreasing phosphorylation of the mTOR (Cui et al., 2021).

Recent studies have confirmed that m6a modification involved in autophagy and regulated the sensitivity of cancer cells to anti-cancer drugs (Paramasivam and Priyadharsini, 2021). RNA-seq shows that m6a modification may induce autophagy activation through stabilizing BECN1 mRNA (Shen et al., 2021). In non-small cell lung cancer cells, METTL3 can positively regulate autophagy through targeting ATG5, ATG7, LC3, and SQSTM1 and thus modulate gefitinib resistance (Liu et al., 2020). Study observed significantly downregulated METTL3 in human sorafenib-resistant HCC and then identified that METTL3-mediated FOXO3 mRNA stabilization was associated with blocked autophagy which enhanced sorafenib resistance of HCC (Lin et al., 2020). Downregulation of METTL14 increased autophagy *via* mTOR signaling pathway, thereby sensitizing pancreatic cancer cells to cisplatin (Kong et al., 2020).

## 3 Autophagy and acute pancreatitis

### 3.1 Impaired autophagy in AP

According to the different modes of material delivery to the lysosomes, three types of autophagy have been described: macroautophagy, microautophagy and chaperone-mediated autophagy (CMA) (Ichimiya et al., 2020). Macroautophagy is studied most deeply and may be the only one detected in normal exocrine pancreas and in pancreatitis (Gukovskaya et al., 2017).

Moreover, differing in the way how autophagosome phagocytoses the degradation targets, non-selective and selective autophagy are described. In the former, cytoplasmic components around the site of autophagosome biogenesis are encapsulated randomly in autophagosomes usually induced by starvation. Nevertheless, in selective autophagy, autophagosomes actively engulf certain substances identified by autophagy proteins, such as mitophagy and ER-phagy. Both selective and non-selective autophagy each seem to be activated in AP (Gukovskaya et al., 2017).

#### 3.1.1 Increased autophagosomes in AP

The significant feature of AP is the accumulation of large vacuoles in acinar cells (Gukovskaya et al., 2017). AP does not block autophagosome formation, but rather stimulates it. TEM and immunogold-TEM studies show that two morphologically different vacuoles were found in AP acinar cells, namely autophagosome, double-membrane vacuoles, containing intact sequestered material, and autolysosome, containing partially degraded substrate. The significantly increased expression of LC3, ATG5 and ATG7 demonstrate that autophagosome is activated and related to vacuoles accumulation in AP acinar cells (Mareninova et al., 2009).

Vacuole membrane protein 1(VMP1) was considered to be related to autophagosome biogenesis in acinar cell. Recent study reveals that observably increased levels of LC3-II and SQSTM1 in VMP1 KO mice, which promote inflammation, acinar-to-ductal metaplasia, and fibrosis in mice pancreas. In addition, loss of acinar cell VMP1 leads to spontaneous pancreatitis in mice through ER stress and activation of the NFE2L2/Nrf2 pathway (Wang et al., 2021a). Furthermore, a study shows that CCK-treated human pancreas slice decreased STX2 levels provoking amylase secretion and autophagic vacuole formation by enhancing Atg16L1/CHC complex assembly (Dolai et al., 2018).

The nuclear translocation of Nrf2 promotes excessive autophagy in severe acute pancreatitis-related acute lung injury through the p62–Keap1–Nrf2 signaling pathway in mice (Kong et al., 2021). Loss of estrogen-related receptor γ (ERRγ) result in mitochondrial dysfunction and further increases autophagosome accumulation and ER stress in pancreatic acinar cells (Choi et al., 2022).

Recently, microRNAs (miRNA) have been proven to regulate the initial stages of autophagy in AP (Yuan et al., 2021). MiR-141 can restrain the formation of autophagosomes in AP through binding to the 3′UTR region of HMGB1, resulting in decreased expression of downstream protein beclin-1 (Zhu et al., 2016). MiR-148a inhibits initial autophagy by down-regulating the interleukin-6 (IL-6)/Signal Transducers and Activators of Transcription 3 (STAT3) signaling pathway (Miao et al., 2019). Additionally, miR-181 b can activate the mTOR/Akt signaling pathway, and then inhibits the expression of beclin-1 and LC3 (Liu et al., 2018a). MiR-155 contributes to the accumulation of autophagosomes by inhibiting Rictor and MAP3K7 binding protein two which negatively regulated Beclin-1 (Wan et al., 2019; Zhang et al., 2020). MiR-375 inhibits autophagy and promotes inflammation and the apoptosis of rat pancreatic acinar cells *via* targeting ATG7 (Zhao et al., 2020). MiR-92b-3p attenuates inflammation and autophagy by targeting TRAF3 and suppressing MKK3-p38 pathway in caerulein-induced AR42 J cells (Sun et al., 2020). ATG7-enhanced impaired autophagy exacerbates AP by promoting regulated necrosis *via* the miR-30b-5p/CAMKII pathway (Ji et al., 2022).

Furthermore, study found that long non-coding RNA FENDRR regulates autophagy through epigenetic suppression of ATG7 by binding PRC2 in AP (Zhao et al., 2021).

#### 3.1.2 Disfunction of lysosomes in AP

The central physiologic function of the pancreatic acinar cell is to synthesize, transport, store and secrete digestive enzymes. Recent studies demonstrate that the functions of lysosomes are deranged in pancreatitis and underlie the mechanisms involved in impaired autophagy of AP. It is widely noticed by TEM that zymogen contents and lysosomal contents locate in a common compartment (Saluja et al., 1987; Saluja et al., 1989). The lysosomes containing cathepsin B fuse with the vacuoles containing trypsin and trypsinogen, and then transform trypsinogen into trypsin. This physiological process relies on a stable lysosomal membrane and sufficient activity of hydrolase (Zhang et al., 2021). Unstable lysosomal vacuoles would rupture and release trypsin and cathepsin B into cytoplasm, thus resulting in apoptosis or necrosis.

Multiple studies show that lysosomes formation decreased in cerulein-treated mouse pancreatic acinar cells according to downregulated LAMP one and LAMP 2, which stabilize lysosomal membrane and protect the cytoplasm from acid hydrolases (Saftig and Klumperman, 2009; Wang et al., 2019). LAMP proteins are protected from decomposition by acid hydrolases due to highly glycosylated molecular structure and relatively stable hydrolases complexes. Research has shown that experimental pancreatitis leads to changes in Cat B maturation which result in cutting of luminal part of LAMP molecule close to the transmembrane domain (Mareninova et al., 2015).

Normal activities of lysosomal hydrolases including cathepsins B and L also decreased in experimental pancreatitis (Mareninova et al., 2009; Gukovsky and Gukovskaya, 2010). The mechanism may be lack of mature cathepsins, accumulation of intermediate forms and the formation of abnormal activity of hydrolase complexes. It has been proved that Cat B transforms trypsinogen into trypsin, while Cat L degrade trypsinogen and trypsin. Cathepsin B-deficient mice do not show pathologic trypsinogen activation in response to caerulein stimulus. The imbalance between enhanced Cat B-mediated conversion of trypsinogen to trypsin and the of inefficient degradation of trypsin and trypsinogen by Cat L may provoke accumulation of trypsin in pancreatitis (Mareninova et al., 2009).

TFEB, a master regulator of lysosomal biogenesis, has been confirmed to be associated with the pathogenesis of experimental pancreatitis (Wang et al., 2019; Wang et al., 2020b). cerulein activated MTOR and increased the levels of phosphorylated TFEB, as well as improving pancreatic proteasome activities that led to accelerated TFEB degradation resulting in decreased number and function of lysosomes in mouse pancreas. It has been proved that store-operated Ca^2+^ entry (SOCE) triggered calcium overload and activated TFEB *via* calcineurin activation, thus promoting transcriptional activation of multiple autophagy-associated genes (Zhu et al., 2018). This indicated interaction between Ca^2+^ signaling pathway and autophagy flux. In addition, food restriction determines the susceptibility of mouse model to coxsackievirus infection and pancreatitis by regulating TFEB and autophagy (Alirezaei et al., 2021).

Study shew that AMPK and SIRT1 were downregulated during AP occurrence and activation of AMPK relieved accumulation of autophagy vacuoles and inhibited inflammation reaction by up-regulating SIRT1 in AP (Wang et al., 2021b).

Galectin-9 binds to Asn^175^ of Lamp2 and poly-LacNAc moieties to maintain lysosome function in highly secretory cells including intestinal Paneth cells and pancreatic acinar cells (Sudhakar et al., 2020). Galectin-9 knockout cells showed more abnormal lysosomes with partial degradation materials, increased accumulation of LC3 and Lamp2, more autophagic vacuoles, and higher lysosomal pH that was associated with impaired lysosomal hydrolase activity. MiR-352 obstructed the autophagy process through targeting the mRNA of LAMP-2 and Cat L1, which resulted in dysfunction of lysosomes and the abnormal activation of trypsin (Song et al., 2018).

Recent study indicates that dysregulation of mannose-6-phosphate (M6P) pathway mediates disorder of lysosome and autophagy and affects cholesterol metabolism (Mareninova et al., 2021). GNPTAB gene that code the key enzyme of M6P pathway regulates the lysosomal system and autophagy in exocrine pancreas. GNPTAB knockout perturbed processing of cathepsins and the maturation of lysosomes, thus diminishing lysosomal proteolytic capacity. In addition, Gnptab deficiency increases total and free cholesterol in acinar cell and result in unbalanced distribution of cholesterol in mitochondria and lysosomes. More interestingly, Gnptab ablation also causes increased levels of serum amylase and lipase, inflammation, as well as parenchymal necrosis of mice pancreas (Mareninova et al., 2021).

Pancreatitis stimuli motivates SNAP23 connection with the STX17 SNARE complex required for autolysosome formation. SNAP23-KD-induced blockade of autophagosome-lysosome fusion by inhibiting SNARE complex which mediates fusion of these two vesicles in experimental pancreatitis rather than physiological starvation. SNAP23-KD prominently disrupted autophagosome STX17 and reduced binding with lysosomal VAMP8 (Dolai et al., 2021).

Mitochondrial dysfunction is an early event in human AP or experimental pancreatitis. The abnormal opening of the permeability transition pore cause the loss of mitochondrial membrane potential (Mukherjee et al., 2016). Furthermore, the activity of F-ATP synthase decreases obviously in AP (Biczo et al., 2018). Cyclophilin D (CypD) was found to be one of the switches of permeability transition pore. Lack of CypD restores the polarity of mitochondrial membrane and positively regulate lysosomal function and autophagic flux in rodent models of pancreatitis (Mukherjee et al., 2016), which indicate the relationship between mitochondria and autophagy pathway.

### 3.2 Genetic and pharmacologic model of autophagy in AP

To explore the association between autophagy, valuable genetic model has been applied in practice. Transgenic green fluorescent protein conjugated LC3 mice (GFP-LC3) is the most classic tool. A recent study compared GFP-LC3 mice with wild-type mice (WT) and found that the expression of GFP-LC3 significantly increased endogenous LC3-II levels in exocrine pancreas by down-regulating the expression of ATG4B, as well as the formation of autophagosome increased 3-fold (Mareninova et al., 2020). However, this physiological interference of GFP-LC3 makes no obvious difference in liver, lung, and spleen.

Gene knockout models also are widely used. Many scholars observed that spontaneous pancreatitis occurs in mice with autophagy pathway related genes ablation including deficiency of ATG5, ATG7 and LAMP (Diakopoulos et al., 2015; Mareninova et al., 2015). Regardless of any level of autophagy flux, homeostasis of acinar cell will be disrupted and then induce spontaneous pancreatitis, manifested as inflammation and fibrosis in gene ablation models (Diakopoulos et al., 2015). It is worth noting that the genetic model itself might have effects on quality of researches.

In addition, pharmacologic inhibitors of autophagy have been widely used to manipulate autophagy. Chloroquine (CQ) increases endogenous LC3-II both in normal pancreas and AP model of mice, thereby regulating basal autophagy of pancreas tissue (Wang et al., 2019). Bafilomycin, a lysosomal inhibition, is usually used to regulate lysosomal functions (Mareninova et al., 2020). However, rare study focuses on if medicine could relieve inflammation of pancreas by autophagy pathway.

### 3.3 Therapy targeting autophagy

Taking intervention from perspectives of autophagy has caught the eyes of scientists in recent years (Table 1). Several autophagy regulators including small-molecule autophagy modulators (rapamycin, wortmannin, chloroquine, and 3-methyladenine), inhibitors of PI3K-AKT-MTOR signaling axis, AMPK activators, lysosomal inhibitors and autophagy-targeting compounds have been discovered and applied to cancer, neurodegenerative and metabolic diseases (Mizushima and Levine, 2020; Kocak et al., 2022). However, the low specificity to autophagy and multiple pharmacological effects of these compounds remained great challenges for scholars. Application of Autophagy regulator in AP is rarely reported. 3-methyladenine, a VPS34 inhibition, decreased the levels of inflammatory cytokines in AP model mice by modulating autophagy flux which is related with the activation of NF-κB signaling pathway and the caspase-1-IL-1β pathway (Mareninova et al., 2020). However, there is rare experimental data or clinical trial in human due to lack of established human pancreatic acinar cell line and accepted methods to separate human primary pancreatic acinar cells from pancreas.

**TABLE 1:** Application of approved autophagy modulators in diseases.

Agents	Targets	Effect on autophagy	Diseases	References
Temsirolimus、Everolimus	MTOR	Enhanced autophagy	Cancer	Kwitkowski et al. (2010), Cives and Strosberg (2018)
Metformin、Resveratrol	AMPK	Enhanced autophagy	Cancer, neurodegenerative diseases	Wang et al. (2018), Pineda-Ramírez et al. (2020)
Chloroquine、Hydroxychloroquine	Lysosomal lumen alkalizer	Suppressed autophagy	Cancer	Boya et al. (2003), Ding et al. (2011)
Azithromycin	50S ribosomal subunit	Suppressed autophagy	Infection	Renna et al. (2011)
Tioconazole	ATG4A/ATG4B	Suppressed autophagy	Cancer	Liu et al. (2018b)
Nicardipine	Calcium channels	Suppressed autophagy	Hypertension	Ochi et al. (2015)

So, it is still unclear whether timely intervention on autophagy could terminate progressive destruction of pancreatic acinar cells and relieve the cascade of inflammatory. To date, establishing a predictive biomarker to monitor autophagy in AP is meaningful for developing new autophagy modulators. In addition, organ specificity also needs to be considered prudently to reduce side effects.

## 4 Summary

Although the roles of autophagy in AP have received more attention of scholars in recent years, the specific mechanism of autophagy flux changes in AP remain unclear. Proper basal autophagy may positively maintain cellular homeostasis, the role of impaired or excessive autophagy in AP is worth exploring further. In addition, current researches have suggested the involvement of epigenetic regulation of autophagy pathway in several diseases. M6a modifications show key roles in modulating autophagy, but few relative studies focus on the epigenetic regulation of autophagy in AP. Elucidating the mechanisms underlying the different stages of autophagic flux dysfunctions will provide us new insights in uncovering potential molecular targets to treat or alleviate the severity of pancreatitis.
